# Increased lengthening frequency does not adversely affect the EOSQ scores in magnetically controlled growing rod surgeries in 133 subjects followed to final fusion

**DOI:** 10.1007/s43390-024-00923-x

**Published:** 2024-07-06

**Authors:** Sheryl Zhi Wen Saw, Jack Zijian Wei, Jason Pui Yin Cheung, Kenny Yat Hong Kwan, Pediatric Spine Study Group, Kenneth Man Chee Cheung

**Affiliations:** 1 Department of Orthopaedics and Traumatology, The University of Hong Kong Shenzhen Hospital, Shenzhen, China; 2 Department of Orthopaedics and Traumatology, The University of Hong Kong, Pokfulam, Hong Kong; 3 Pediatric Spine Foundation, Valley Forge, PA USA

**Keywords:** Magnetically controlled growing rods, MCGR, Early-onset scoliosis, Rod lengthening, EOSQ, Growing rods

## Abstract

**Purpose:**

Magnetically Controlled Growing Rod (MCGR) allows frequent outpatient rod lengthening when treating Early Onset Scoliosis (EOS) patients. But there is lack of expert consensus on the optimal MCGR lengthening interval. EOS 24-Item Questionnaire (EOSQ) is validated for assessing health-related quality of life (HrQOL), family burden, and satisfaction. This is the first study assessing how MCGR lengthening intervals affects patient-perceived outcomes.

**Methods:**

This is a multicentred cohort study with subjects recruited from 2012 to 2018 and followed till fusion. EOS subjects who underwent MCGR surgeries were grouped into high, medium or low lengthening interval subgroups based on 16 and 20 week cut-offs. Repeated measure analysis was performed on EOSQ’s specified 12 domains. EOSQ results were taken: before index surgery, after index surgery, and prior to definitive treatment. Demographic, clinical and radiographic data were included in model adjustment.

**Results:**

133 subjects with mean follow-up of 3.5 (± 1.3) years were included, with 60 males and 73 females; 45 idiopathic, 23 congenital, 38 neuromuscular, and 27 syndromic patients. Mean Cobb angle at surgery was 67° (± 22°) with mean age of 8.3 (± 2.5) years. Between groups, clinical and radiographic parameters were comparable. Higher EOSQ scores in medium lengthening interval subgroup was present in fatigue (*p* = 0.019), emotion (*p* = 0.001), and parental impact (*p* = 0.049) domains, and overall score (*p* = 0.046). Trendline contrast between subgroups were present in general health (*p* = 0.006) and physical function (*p* = 0.025) domains.

**Conclusion:**

Patient-perceived outcome improvements appear similar between lengthening interval subgroups. All MCGR lengthening intervals were tolerated by patients and family, with no negative impact observed.

**Level of evidence:**

Prognostic Level III.

## Introduction

Early-Onset Scoliosis (EOS) is a condition determined by a lateral deviation of the spine in children below ten years old, often diagnosed based on having a Cobb angle of 10° or above. If left untreated, the condition may exacerbate until the completion of puberty, adversely affecting the child’s pulmonary and physical function as well as cosmetic appearance. Children diagnosed with severe EOS (Cobb angle > 40°) are often offered growth-friendly surgical treatments (i.e. distractible spinal implants) if prior conservative treatments have failed to control spinal curve progression [1, 2]. Recent advancements in distractible spinal implants, i.e. Magnetically Controlled Growing Rod (MCGR), allows frequent lengthening of the implanted rod in an outpatient setting via remotely controlled external magnets [3, 4], which is also shown to be successfully monitored without radiation [5, 6]. The MCGR is proven to be clinically safe and effective, with successful control of spinal deformity progression in long-term follow-up while promoting spinal growth [7–12]. Additionally, not only does the MCGR help alleviate detrimental effects of traditional treatments, overcoming needs for repeated surgeries and general anaesthesia, which often leads to increased risks of infection, but it has also helped lower patients’ socioeconomic burden [11, 13, 14].

Nonetheless, various studies have noted that guidelines regarding the MCGR treatment have yet to be completely established, especially regarding protocols on MCGR rod distraction intervals [7, 15–17]. Not only is the ideal MCGR distraction technique not established, but the rod lengthening procedure is also shown to have potential pitfalls such as rod slippage and law of diminishing returns, deeming the need for further investigation [17–20]. The lack of expert consensus on an optimal distraction interval not only leads to lack of standardized practice between different countries and centres, but it has also evoked disputes on whether current practices allows achievement of optimal holistic treatment outcomes. Additionally, although recent preliminary studies began evaluating optimal distraction intervals, with suggested intervals dispersedly ranging from 1 to 6 months, outcome measures investigated were mainly focused on clinical and radiographic parameters, neglecting the importance of patient-perceived outcomes (i.e. health-related quality of life (HrQOL), family burden, patient satisfaction) [15, 20–22]. Even though the validated measure of EOS 24-Item Questionnaire (EOSQ) has been established for assessing HrQOL, family burden and satisfaction in patients with EOS [23, 24], limited studies have validated MCGR protocol based on EOSQ scores.

Given the lack of consensus on an optimal MCGR distraction interval, this study aims to be the first to investigate correlations between MCGR distraction intervals and patient-perceived outcomes in EOS patients using EOSQ scores. Provided that frequent hospital visits needed in traditional growing rod treatments have adversely affected patients’ HrQOL [25, 26], it is hypothesized that reducing MCGR lengthening intervals (i.e. increased frequency of lengthening) would adversely affect patients’ HrQOL, with increased family burden and reduced satisfaction.

## Materials and methods

The analysis consists of retrospectively collected registry data from the multicentred PSSG research database. The database included demographic, clinical, and radiological data on children diagnosed with EOS, who had a MCGR implanted between 2012 and 2018, and had more than 3 questionnaire responses recorded by their parents, with minimum 2 year follow-up. Local ethics review board approval was obtained prior to the study’s commencement and data was recorded as standard-of-care protocol in included sites.

Patients who underwent MCGR surgery with completed records of EOSQ, clinical data and radiographic data (1) prior to MCGR implant surgery, (2) after MCGR implant surgery, and (3) prior to completion of MCGR treatment, were included in the study. Patients were also only included in the study if they had definitive treatment completion (i.e. fusion, in-situ rod retention, rod removal). Those who did not undergo MCGR distraction, or had ongoing treatment were also excluded.

Each patients’ average MCGR lengthening interval was calculated via dividing their treatment duration by the total number of distractions during the treatment period. For the main analysis, the cohort was divided into three subgroups based on available data and current clinical trends of MCGR application: Low lengthening interval (LLI), medium lengthening interval (MLI) and high lengthening interval (HLI) subgroups. The LLI subgroup included subjects with a lengthening interval below 16 weeks, MLI subgroup included those with a lengthening interval from 16 to 20 weeks, and HLI subgroup included those with a lengthening interval of over 20 weeks.

EOSQ scores were analysed based on its specified 12 domains in 3 divisions: (1) HrQOL (8 domain): general health, pain/discomfort, pulmonary function, transfer, physical function, daily living, fatigue/energy level, emotion; (2) family burden (2 domains): parental impact, financial impact; (3) treatment satisfaction (2 domains): child satisfaction, parent satisfaction. The score of each domain is averaged from a selection of 5-point scaled questions and calculated out of a 100 points scale, where a score of 100 indicates the best treatment outcomes. Number of questions in each domain varied from 1 to 5 questions. Mean scores were calculated for each of the 3 divisions and overall 12 domains. Differences in domain, division, and overall scores between subgroups were examined at three timepoints: (1) prior to index surgery (Pre-index), (2) after index surgery (Post-index), and (3) prior to definitive treatment indicating MCGR treatment completion (Pre-definitive). If patients had multiple EOSQs done between timepoints, the EOSQ taken closest to each timepoint was used for analysis.

Confounder analysis considered all available demographic, clinical and radiographic data. Demographic data included: age (year), gender, race, EOS classification (C-EOS), ASA scaled comorbidities, prior treatment (i.e. bracing, casting, VBT, TGR, Shilla). Clinical data included: weight (kg), height (cm), body mass index (BMI) (kg/m^2^), ambulatory status, index surgery type (i.e. conversion vs non-conversion). Radiographic data included: spine height (total spine height measured using X-ray from T1-S1) (cm), and major Cobb angle (°). Patients were recruited from 44 centres and had a follow-up period up till November 2021.

### Statistical analysis

Statistical analysis was performed using IBM SPSS Statistics, Version 28 (IBM Corp., Armonk, N.Y., USA). Normality of lengthening interval data was assessed via skewness and kurtosis. Standard Deviation (SD) was indicated for recorded values. Statistical significance was set at *p* < 0.05.

Variability in demographics and clinical conditions were proven to affect EOSQ scores [27, 28], hence to determine the impact of potential confounding variables, One-way ANOVA and Chi-squared analysis were performed for baseline demographic, clinical and radiographic data between LLI, MLI and HLI subgroups. Pearson correlation was then performed between confounding variables and EOSQ scores at the three timepoints. Correlations between pre-index EOSQ scores and lengthening intervals were assessed in the 12 domains.

Repeated measures analysis of variance (ANOVA) was adopted to determine the differences in EOSQ scores between LLI, MLI and HLI subgroups. Model adjustment was conducted based on the significant confounding variables from previous Pearson correlation analysis. Domains with significant results in repeated measures ANOVA, had further univariate analysis performed to compare pre-index EOSQ scores between the three subgroups, allowing better understanding of the different time-points’ contribution to the significant outcomes.

## Results

### Descriptive analysis

133 patients were included in the cohort, with 60 (45.1%) males and 73 (54.9%) females; 45 (33.8%) idiopathic, 23 (17.3%) congenital, 38 (28.6%) neuromuscular and 27 (20.3%) syndromic scoliosis patients. 94 (70.7%) patients were Caucasians, 25 (18.8%) patients were non-Caucasian, and 14 (10.5%) patients’ race was unknown. Index MCGR surgeries were performed at a mean age of 8.3 (± 2.5) years with a mean Cobb angle of 67° (± 22°). The mean treatment duration was 3.5 (± 1.3) years, with a mean of 9.1 (± 4.8) MCGR distractions conducted at a mean interval of 23.3 (± 11.8) weeks.

Analysis of the cohort’s lengthening interval showed a kurtosis of 8.0 and skewness of 2.6, indicating deviation from the normal distribution. The three lengthening interval subgroups: LLI subgroup (< 16.0 weeks, *n* = 21), MLI subgroup (16.0 to 20.0 weeks, *n* = 52), and the HLI subgroup (> 20.0 weeks, *n* = 60), had mean lengthening intervals of 13.9 weeks (± 2.2 weeks), 17.9 weeks (± 1.1 weeks), and 31.4 weeks (± 13.6 weeks), respectively. One-way ANOVA indicated significant difference in mean values between subgroups (*t*(2) = 43.2, *p* < 0.001). Mean treatment duration of LLI (3.6 years ± 1.6 years), MLI (3.5 years ± 1.4 years), and HLI (3.4 years ± 1.1 years) subgroups were comparable. Mean number of distractions for LLI, MLI, and HLI subgroups were 13.9 (± 6.4), 10.3 (± 4.0), and 6.3 (± 2.6), respectively.

Baseline variables of race (Caucasian vs non-Caucasian) and prior treatment with cast showed significant differences between subgroups. These variables were adjusted as confounders throughout the investigation. No other significant differences were detected in pre-index demographic (index surgery age, gender, aetiology, comorbidity, prior treatment), clinical (height, weight, BMI, ambulatory status, index surgery type), and radiographic (Cobb angle, spine height) variables between the subgroups (Table 1). Correlations between pre-index EOSQ scores and lengthening interval were not statistically significant in Pearson correlation analysis for all domains.

**Table 1:** Baseline subgroup demographics

Variable	Low lengthening interval	Medium lengthening interval	High lengthening interval	Significance (*p* value)
**Demographics**				
Total sample size	21 (15.8%)	52 (39.1%)	60 (45.1%)	–
Age at index surgery (year)	7.5 (± 2.4)	8.0 (± 2.5)	8.8 (± 2.4)	0.069
Gender				
Male	9 (42.9%)	26 (50.0%)	25 (41.7%)	0.660
Female	12 (57.1%)	26 (50.0%)	35 (58.3%)	
Race (Caucasians vs non-Caucasians)				
Asian	0 (0.0%)	0 (0.0%)	2 (3.3%)	0.024
Black	1 (4.8%)	5 (9.6%)	13 (21.7%)	
Caucasian	18 (85.7%)	44 (84.6%)	32 (53.3%)	
Others	1 (4.8%)	2 (3.8%)	1 (1.7%)	
Unknown	1 (4.8%)	1 (1.9%)	12 (20.0%)	
Aetiology (idiopathic vs non-idiopathic)				
Idiopathic	9 (42.9%)	19 (36.5%)	17 (28.3%)	0.418
Congenital	5 (23.8%)	8 (15.4%)	10 (16.7%)	
Neuromuscular	4 (19.0%)	14 (26.9%)	20 (33.3%)	
Syndromic	3 (14.3%)	11 (21.2%)	13 (21.7%)	
Comorbidities (ASA level)				
ASA low level (I and II)	9 (42.9%)	14 (26.9%)	20 (33.3%)	0.409
ASA high level (III and IV)	12 (57.1%)	38 (73.1%)	40 (66.7%)	
Prior treatment				
Yes	11 (52.4%)	29 (55.8%)	32 (53.3%)	0.952
No	10 (47.6%)	23 (44.2%)	28 (46.7%)	
Prior treatment type-brace				
Yes	3 (14.3%)	13 (25.0%)	20 (33.3%)	0.218
No	18 (85.7%)	39 (75.0%)	40 (66.7%)	
Prior treatment type-cast				
Yes	7 (33.3%)	7 (13.5%)	6 (10.0%)	0.033
No	14 (66.7%)	45 (86.5%)	54 (90.0%)	
Prior treatment type-VEGF/TGR/Shilla				
Yes	4 (19.0%)	4 (7.7%)	6 (10.0%)	0.353
No	17 (81.0%)	48 (92.3%)	54 (90.0%)	
Prior treatment type-others				
Yes	0 (0.0%)	1 (1.9%)	4 (6.7%)	0.258
No	21 (100.0%)	51 (98.1%)	56 (93.3%)	
**Clinical data**				
Pre-index height (cm)	120.0 (± 16.1)	124.4 (± 17.9)	124.5 (± 15.9)	0.560
Pre-index weight (kg)	25.3 (± 9.9)	25.9 (± 9.3)	27.4 (± 10.1)	0.614
Pre-index BMI (kg/m^2^)	16.9 (± 3.5)	16.4 (± 2.9)	17.5 (± 4.2)	0.316
Pre-index ambulatory status				
Yes	17 (81.0%)	44 (84.6%)	45 (75.0%)	0.446
No	4 (19.0%)	8 (15.4%)	15 (25.0%)	
Index surgery type (conversion vs no conversion)				
Conversion	7 (33.3%)	17 (32.7%)	28 (46.7%)	0.268
No conversion	14 (66.7%)	35 (67.3%)	32 (53.3%)	
**Radiographic data**				
Pre-index major cobb angle (degree)	68 (± 18)	67 (± 21)	66 (± 24)	0.936
Pre-index spine height (cm)	27 (± 5)	30 (± 7)	29 (± 6)	0.259

*ASA* american society of anaesthesiologist*, LLI* low lengthening interval*, MLI* medium lengthening interval*, HLI* high lengthening interval

### Repeated measures ANOVA

Significant differences in absolute EOSQ scores between lengthening interval subgroups were noted in 2 domains of the HrQOL division: fatigue (*F*(1,2) = 4.2, *p* = 0.019) and emotion (*F*(1,2) = 7.1, *p* = 0.001), 1 domain in the family burden division: parental impact (*F*(1,2) = 3.1, *p* = 0.049), and overall EOSQ score (*F*(1,2) = 3.2, *p* = 0.046). Trendline contrasts also showed significant differences in two domains of the HrQOL division: general health (*F*(1,2) = 5.3, *p* = 0.006) and physical function (*F*(1,2) = 3.8, *p* = 0.025) (Table 2).

**Table 2:** Repeated measures ANOVA results showing the effects of MCGR lengthening interval on EOSQ scores at pre-index, post-index, and pre-definitive timepoints

EOSQ domain	*F*	Significance (*p* value)	Observed power	Effect size
**HrQOL**				
Between groups	1.6	0.200	0.34	0.03
Within groups	2.0	0.100	0.59	0.04
Contrast	2.0	0.139	0.41	0.04
General health				
Between groups	0.6	0.563	0.14	0.01
Within groups	4.7	0.001	0.95	0.08
Contrast	5.3	0.006	0.83	0.09
Pain and discomfort				
Between groups	1.7	0.181	0.36	0.04
Within groups	1.4	0.238	0.43	0.03
Contrast	2.1	0.124	0.17	0.02
Pulmonary function				
Between groups	1.0	0.367	0.22	0.02
Within groups	1.1	0.367	0.34	0.02
Contrast	0.3	0.768	0.09	0.01
Transfer				
Between groups	2.5	0.090	0.49	0.05
Within groups	1.7	0.160	0.51	0.03
Contrast	1.2	0.299	0.26	0.02
Physical function				
Between groups	2.5	0.084	0.50	0.05
Within groups	1.9	0.112	0.57	0.04
Contrast	3.8	0.025	0.68	0.07
Daily living				
Between groups	0.4	0.669	0.11	0.01
Within groups	2.1	0.077	0.63	0.04
Contrast	2.0	0.135	0.41	0.04
Fatigue				
Between groups	4.2	0.019	0.72	0.08
Within groups	1.7	0.147	0.52	0.04
Contrast	2.4	0.097	0.42	0.05
Emotion				
Between groups	7.4	0.001	0.93	0.13
Within groups	0.4	0.780	0.15	0.01
Contrast	0.8	0.452	0.18	0.02
**Family burden**				
Between groups	1.2	0.293	0.27	0.02
Within groups	0.9	0.463	0.27	0.02
Contrast	1.0	0.381	0.22	0.02
Parental impact				
Between groups	3.1	0.049	0.59	0.06
Within groups	1.6	0.173	0.49	0.03
Contrast	0.6	0.535	0.47	0.04
Financial impact				
Between groups	0.2	0.790	0.09	0.00
Within groups	0.4	0.800	0.15	0.01
Contrast	0.5	0.607	0.13	0.01
**Satisfaction**				
Between groups	2.3	0.104	0.46	0.04
Within groups	0.6	0.657	0.20	0.01
Contrast	0.7	0.494	0.17	0.01
Parent satisfaction				
Between groups	1.8	0.176	0.36	0.03
Within groups	0.4	0.817	0.14	0.01
Contrast	0.3	0.750	0.10	0.01
Child satisfaction				
Between groups	2.0	0.136	0.41	0.04
Within groups	0.8	0.518	0.26	0.02
Contrast	1.2	0.294	0.27	0.02
**Overall**				
Between groups	3.2	0.046	0.60	0.06
Within groups	1.4	0.240	0.43	0.03
Contrast	1.6	0.201	0.24	0.02

*MCGR* magnetically controlled growing rod*, EOSQ* early onset scoliosis questionnaire*, HrQOL* health-related quality of life

The MLI subgroup was found to have both a higher absolute EOSQ scores and a positive trend towards improvement by the end of EOSQ treatment in the five aforementioned domains and overall score (effect size: 0.06–0.13, observed power: 0.59–0.93) when compared to the LLI (mean difference: 8.8 ± 5.4) and HLI subgroups (mean difference: 8.0 ± 3.5). In contrast, although not statistically significant, the LLI subgroup showed no improvement in most EOSQ scores, while the HLI subgroup had mixed results. A tendency of higher scores and positive trendline towards improvement in the MLI subgroup was observed in the remaining six domains and three subdivisions. Repeated measures model for each EOSQ domain was selectively adjusted based on detected confounders (*p* < 0.05), including ambulatory status, aetiology, prior treatment, complications, and parameters involving major cobb angle, height and weight (Appendix 1).

### Other analysis

Further univariate analysis of the four domains with significant differences in absolute EOSQ scores in the repeated measures analysis suggested that significant differences between lengthening interval subgroups were accounted for at pre-index or post-index surgery time points for two of the domains: fatigue (*p* = 0.027) and emotion (*p* = 0.013) (Table 3).

**Table 3:** Univariate analysis of pre-index and post-index EOSQ scores between LLI, MLI and HLI subgroups in domains with significant results in repeated measures analysis

EOSQ domain	*F*	Significance (*p* value)	Observed power	Effect size
Fatigue	3.8	0.027	0.67	0.08
Emotion	4.6	0.013	0.76	0.09
Parental impact	2.6	0.076	0.52	0.04
Overall	2.0	0.143	0.04	0.40

*EOSQ* early onset scoliosis questionnaire*, LLI* low lengthening interval*, MLI* medium lengthening interval, *HLI* high lengthening interval

Patients’ ambulatory status impacted EOSQ score differences most when evaluating effect sizes of confounders in general health domain (*ηp*^2^ = 0.09), parental impact domain (*ηp*^2^ = 0.17), and overall scores (*ηp*^2^ = 0.21). The financial impact domain and family burden division was shown to improve over time irrespective of subgroups, where the financial impact domain improved by a mean score of 7.4 (*p* = 0.023) while the family burden division improved by a mean score of 5.9 (*p* = 0.006). No other significant trends were detected between subgroups in remaining domains.

## Discussion

Various studies have indicated a lack of expert consensus on the optimal MCGR distraction interval. Application of lower lengthening intervals have been shown to mimic physiological growth better, while longer lengthening intervals were preferred by some physicians. Patients’ convenience was cited as a major concern, since long travelling distances were required for patients and families in some countries, such that patients’ HrQOL, family burden and satisfaction were adversely affected by frequent visits [29]. However, infrequent visits also poses increased risks of achieving non-optimal spine lengthening due to decreased MCGR lengthening efficacy over time, ultimately affecting patient’s overall treatment outcome.

This is the first study to demonstrate that all MCGR lengthening intervals shows no adverse effect on EOSQ scores. The strength of this study is that a multi-centred, world-wide registry database was used, where subjects were followed to final fusion with a mean follow-up duration of 3.5 years.

In the longitudinal analysis, notable differences were evident when comparing the MLI subgroup with the HLI and LLI subgroups’ mean EOSQ scores. The MLI subgroup had significantly higher absolute scores in the fatigue, emotion, parental impact domains, and overall score, accompanied with significant trendline contrasts in the general health and physical function domain when compared to other subgroups. However, as indicated by the univariate analysis, two out of these four domains (fatigue, emotion) showed significant absolute EOSQ score differences at the pre- or post-index timepoints. Various confounders also deemed a significant portion of the effect size, suggesting that overall effects cannot be concluded to be definitively attributed to the use of a medium lengthening interval. Overall results suggests that while a medium lengthening interval may evoke trends towards improvement in EOSQ scores, lower or higher lengthening intervals does not correlate with worsened patient-perceived outcomes nor detriment patient’s HrQOL either, with no significant negative trends observed in either subgroup’s EOSQ scores when approaching definitive treatment. Preference for the medium lengthening interval can be attributed to achieving a balance between limiting disruption to patients’ daily lives and sufficient follow-up to attain optimal functional outcomes. Moreover, patient’s financial impact and family burden was shown to have improvement over time towards definitive treatment irrespective of their MCGR lengthening interval. This aligns with our expectations as patients’ and family members’ contentment may naturally improve with treatment success.

Additionally, while some studies suggested that patients were surprisingly compliant with frequent outpatient visits of 3–4 months [3, 30], abiding with the inherent benefits of the MCGR treatment reducing the need for in-hospital stays [3, 29], our study suggests that slightly longer lengthening intervals may also be favoured by patients, with a more tolerable disruption to their daily lives, thus aiding normal daily living resumption, improving their long term HrQOL.

Additionally, several studies have been done based on cohorts with an average lengthening interval of 12–16 weeks [1, 30, 31], likely based on the synchronous benefits of achieving improved clinical and radiological outcomes. Nonetheless, additionally assessed HrQOL outcomes in these cohorts were generally heterogenous, with some suggesting improvements of certain EOSQ domains [30], while others suggesting invariable HrQOL scores throughout the MCGR treatment [32]. In coherence with previous studies, while our study suggests slightly longer lengthening intervals may allow better improvement trends of HrQOL, overall results supports the observation that patients have no negative perception towards any out-patient rod distractions intervals. Given that previous studies have suggested a 13 week MCGR lengthening interval for optimal radiological and clinical outcomes, where any intervals below it would increase the risk of re-operation or proximal junctional kyphosis and any longer intervals would impede optimal spine height gain [21, 33, 34], our study postulates the idea that simultaneous achievement of multi-fold treatment outcomes can be accomplished, where maintenance of an improved HrQOL and decreased family burden can be achieved concomitantly.

Nevertheless, as indicated by various studies assessing MCGR treatment outcomes, a minimal clinically important difference (MCID) for EOSQ has yet to be defined by experts [25, 35, 36]. Although Shaw et al. and Campbell et al. suggested a 10–20% change in EOSQ score as the MCID [35, 37], their parameter choice was not statistically or clinically justified. Hence, given the lack of normative EOSQ data for baseline comparison, along with the limited observed power, clinical significance of detected differences in EOSQ scores among subgroups in this study cannot be affirmed either.

Despite considering a diverse range of confounders, various important aspects of distraction technique used (e.g. Distraction amount, type of distraction), patient’s socio-economic status, such as income and distance travelled to receive treatment, and centres’ different preferred practices (e.g. Consideration of patients’ preferred lengthening frequency), are also potential confounders not obtained in this study. Although till date this study involves the largest cohort for investigating correlations between MCGR lengthening interval and patient oriented outcomes, since all 133 patients in the study were collected from North American centres, with 131 from the United States, and 2 from Canada, this study may better represent patients’ HrQOL in Northern America in comparison to patients from other regions.

To conclude, although the MCGR has been widely implemented for over 10 years [3], compared to other traditional methods, it remains to be a new procedure requiring further protocol standardization. This study assists the determination of an optimal MCGR lengthening interval and, for the first time, demonstrated effects of different MCGR lengthening intervals on patient-perceived treatment outcomes: HrQOL, family burden, and satisfaction. Irrespective of MCGR lengthening intervals, no significant negative impact on patient-perceived outcomes were observed as assessed by the EOSQ. Despite the limitations, apparent differences noted in this study warrant the necessity for further investigation into this topic, while encouraging lengthening schedules to be adjusted based on clinical and radiological outcome, given the tolerance indicated by patients and their families irrespective of lengthening interval chosen.

## Data Availability

Datasets generated and analyzed in the current study are available from the corresponding author upon request.

## Appendix

See Table 4.

**Table 4:** Significant confounders for each EOSQ domain

EOSQ Domain and confounders	*F*	Significance (*p* value)	Observed power	Effect size
**HrQOL**				
Pre-definitive ambulatory status	41.3	< 0.001	1.00	0.27
Pre-index major cobb angle	8.1	0.005	0.81	0.07
General health				
Pre-definitive ambulatory status	10.9	0.001	0.90	0.09
Pain & discomfort				
Pre-index major cobb angle	5.1	0.027	0.61	0.06
Weight change (pre-index to pre-definitive)	5.6	0.020	0.65	0.06
Weight change (post-index to pre-definitive)	4.3	0.042	0.53	0.05
Complications number (pulmonary related)	7.7	0.007	0.78	0.08
Pulmonary function				
Post-index major cobb angle	9.7	0.002	0.87	0.09
Cobb angle change (post-index to pre-definitive)	7.6	0.007	0.78	0.07
Transfer				
Pre-index ambulatory status	16.5	< 0.001	0.98	0.14
Pre-definitive height	4.2	0.042	0.53	0.04
Physical function				
Prior treatment (others)	5.3	0.024	0.63	0.05
Cobb angle change (pre-index to post-index)	5.2	0.025	0.62	0.05
Complication number (pulmonary)	9.6	0.002	0.87	0.09
Daily living				
Aetiology (idiopathic vs non-idiopathic)	10.6	0.002	0.90	0.09
Pre-index ambulatory status	66.2	< 0.001	1.00	0.39
Pre-index weight	4.9	0.029	0.59	0.04
Fatigue				
Aetiology (idiopathic vs non-idiopathic)	6.8	0.011	0.73	0.07
Pre-index ambulatory status	5.1	0.027	0.60	0.05
Height change (pre-index to pre-definitive)	6.4	0.013	0.70	0.07
Emotion				
Prior treatment (others)	4.6	0.034	0.57	0.05
Pre-definitive major cobb angle	7.9	0.006	0.79	0.07
Weight change (pre-index to pre-definitive)	6.9	0.010	0.74	0.07
**Family burden**				
Pre-definitive ambulatory status	11.4	0.001	0.92	0.09
Complication number	8.3	0.004	0.83	0.07
Parental impact				
Pre-definitive ambulatory status	21.6	< 0.001	1.00	0.17
Complication number	5.8	0.018	0.67	0.05
Pre-index major cobb angle	5.1	0.026	0.61	0.05
Financial impact				
Complication number	6.3	0.014	0.70	0.05
**Satisfaction**				
Pre-definitive ambulatory status	23.1	< 0.001	1.00	0.18
Pre-definitive major cobb angle	6.7	0.011	0.73	0.06
Complication (pulmonary related)	13.1	< 0.001	0.95	0.11
Complication (implant related)	4.5	0.035	0.56	0.04
Parent satisfaction				
Pre-definitive ambulatory status	16.6	< 0.001	0.98	0.13
Pre-definitive major cobb angle	5.1	0.025	0.61	0.05
Complication (pulmonary related)	8.9	0.004	0.84	0.08
Complications (implant related)	4.7	0.033	0.57	0.04
Child satisfaction				
Pre-definitive ambulatory status	22.2	< 0.001	1.00	0.17
Pre-definitive major cobb angle	10.0	0.002	0.88	0.08
Complications (pulmonary related)	12.0	< 0.001	0.93	0.10
**Overall**				
Pre-definitive ambulatory status	28.7	< 0.001	1.00	0.21
Pre-definitive major cobb angle	15.8	< 0.001	0.98	0.13
Comorbidities (ASA level)	4.8	0.031	0.58	0.04

*EOSQ* Early onset scoliosis questionnaire*, HrQOL* Health-related quality of life*, ASA* American society of anaesthesiologist
